# GENDER DIFFERENCES IN THE ASSOCIATION BETWEEN FRAILTY AND HOSPITALIZATION AMONG OLDER ADULTS IN SOUTH KOREA

**DOI:** 10.1093/geroni/igae098.2763

**Published:** 2024-12-31

**Authors:** Bokyoung Choi, Yumi Shin, Chang-O Kim, Ahreum Choi, Eunhee Choi, Hyeyeon Lee, Soog-nang Jang

**Affiliations:** Institute for Community Care and Health Equity, Seoul, Republic of Korea; Daegu University, Gyeongsan, Republic of Korea; Institute for Community Care and Health Equity, Seoul, Republic of Korea; Institute for Community Care and Health Equity, Seoul, Republic of Korea; Institute for Community Care and Health Equity, Seoul, Republic of Korea; Institute for Community Care and Health Equity, Seoul, Republic of Korea; Chung-Ang University, Seoul, Republic of Korea

## Abstract

This study aimed to investigate the association between frailty and hospitalization experience among older adults in South Korea, while also exploring potential gender differences in this association. We used data from the Korean Longitudinal Study of Aging (2018-2020). Frailty was assessed using the frailty instrument (weakness of grip strength, exhaustion, and social isolation) and divided into three groups: non-frail, pre-frail, and frail. Experience of hospitalization over the past two years was measured by self-reported. Multivariate logistic regression model was applied to examine the association between frailty and hospitalization experience after adjusting for potential confounders including age, education level, and physical activity. Among aged 65 and over older adults in South Korea, the prevalence of frailty was 14.4% (N=656), and the prevalence of hospitalization over the past two years was 9.6% (N=437). Compared to the non-frail group, the frail group had a statistically significantly higher risk of hospitalization (pre-frail OR:1.63 95% CI:1.28-2.08, frail OR:2.50 95% CI:1.85-3.39) after adjusting potential confounders. However, after stratification by gender, the relationship between frailty and hospitality was still statistically significant among older women (pre-frail OR:2.00 95% CI:1.45-2.75, frail OR:3.27 95% CI:2.22-4.80), but not in men. These findings suggest that understanding gender differences in the association between frailty and hospitalization would be beneficial for reducing the care burden among older adults in South Korea. Tailoring interventions and healthcare services to address the specific needs of frail older individuals, particularly focusing on gender-specific differences, may help in preventing hospitalizations and improving overall well-being.

